# The complete chloroplast genome sequence and phylogenetic analysis of *Asplenium antiquum* Makino 1929, an Endangered species in Korea

**DOI:** 10.1080/23802359.2024.2356139

**Published:** 2024-05-20

**Authors:** I-Jin Choi, Ho Jun Joh, Wan-Hee Lee, Dae-Sung Kim

**Affiliations:** Plant Management & Research Division, Seoul Botanical Park, Seoul, Republic of Korea

**Keywords:** *Aspleniaceae*, *asplenium antiquum*, chloroplast genome, phylogenetic relationships, plastome

## Abstract

*Asplenium antiquum* Makino 1929 is one of the Endangered endemic species on the Korean Peninsula. The complete chloroplast of *A. antiquum* is 150,690 bp in length with typical quadripartite structure comprised of large single-copy region of (83,166 bp), a small single copy region (21,932 bp), and two inverted repeat regions, each 22,796 bp in length. 114 genes were detected in the chloroplast genome of *A. antiquum*, comprising 84 protein-encoding genes, 26 tRNA genes, and 4 rRNA genes. The phylogenetic analysis revealed a monophyletic relationship, placing *A. antiquum* as a sister to voth *A. Prolongatum and A. nidus,* forming a subclade of *Asplenium* species within the Aspleniaceae family. The genomic data obtained from this study will serve as valuable information for the species’ genetic classification of *Asplenium.*

## Introduction

*Asplenium antiquum* Makino 1929, a temperate evergreen fern species, is distributed in East Asia. A global phylogeny of Asplenium published in 2020 (Xu et al. 2020) utilized *Asplenium* species from various East Asia regions and divided the genus into eleven clades, which have given informal names pending further taxonomic study. *Asplenium antiquum* belongs to the ‘Neottopteris clade,’ members of which generally have somewhat leathery leaf tissue (Kang 2009). *Asplenium antiquum* is found on Seop Island near Jeju Island and is classified as an Endangered species in Korea. It is essential to secure genetic information that can be distinguished this species from those of other Asian countries. A study by Xu et al. (2020) utilized phylogenetic studies based on partial plastid sequence, which might resolve the subclades of *Asplenium* species. To this date, few studies have reported on the chloroplast genome of *Asplenium* species, including *A. antiquum*. Here, we report the complete chloroplast genome sequence of *A. antiquum* to facilitate taxonomy and phylogenetic approaches from phylogenetic inference on the *Asplenium* genus for future studies.

## Materials and methods

**Figure 1.**
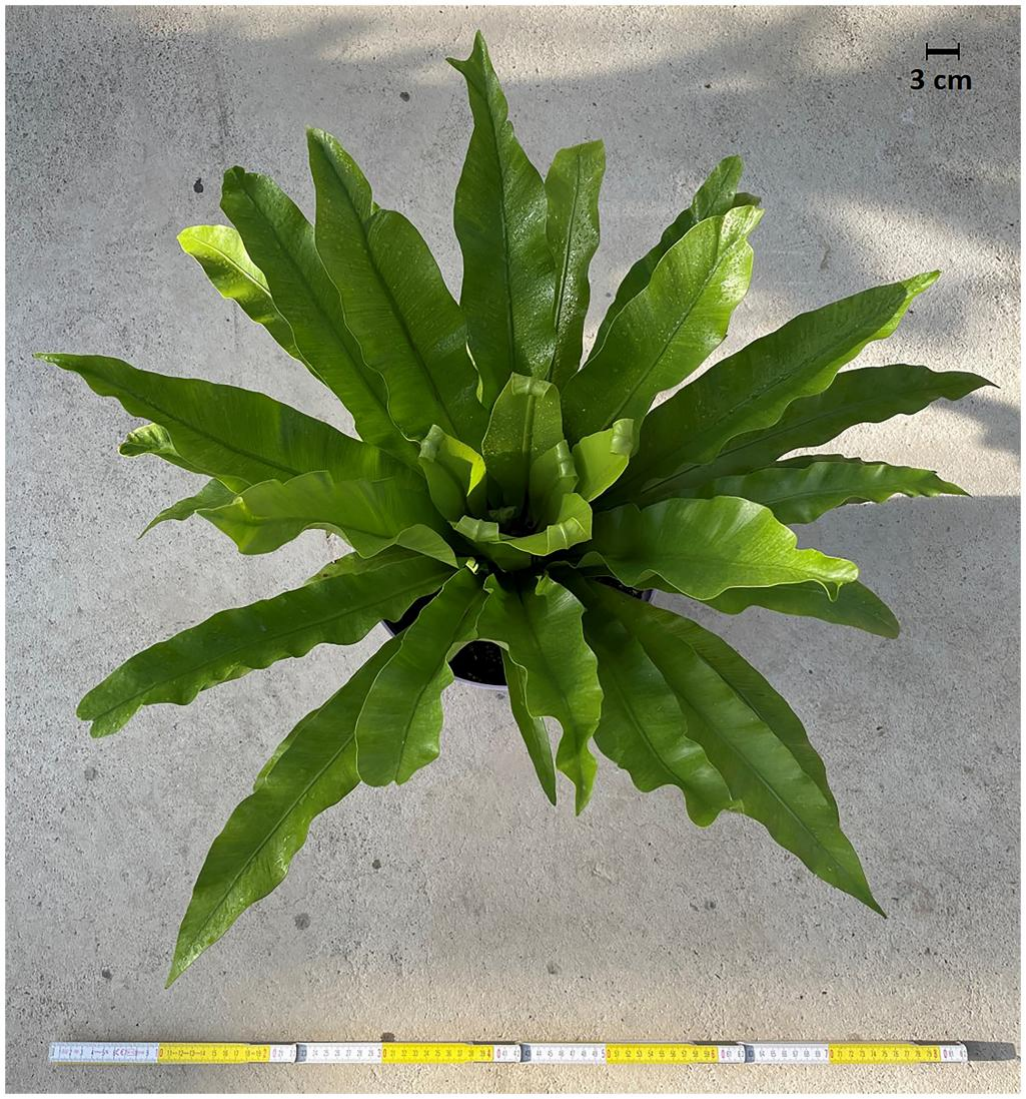
Photograph of *A. antiquum* makino (this photograph was taken by Ho jun joh). The foliage of *A. antiquum* has bright green, arching blades with a pointed end and a strong midrib. The leaves’ total length is 80–100 cm, and their width is 7–12 cm. The lateral veins are obliquely upward.

The species *Asplenium antiquum* Makino specimen was collected from a transferred plant species from Key-ChungSan Botanical Garden (36° 02′ 34"N to 36° 50′ 17"N, 128° 59′ 20"E to 129° 34′ 57"E), Pohang, Republic of Korea. The plant was artificially cultivated in 1988 at Key-ChungSan Botanical Garden for conservation and proliferation purposes. A specimen was deposited at Seoul Botanical Garden (https://botanicpark.seoul.go.kr/front/main.do; contact: I-Jin Choi, ijin2080@seoul.go.kr), under the voucher number 202300394 (Figure 1). Genomic DNA was isolated from approximately 0.2 g of the fresh leaf material and extracted using an i-genomic Plant DNA Extraction Kit (iNtRon biotechnology, South Korea), and the library was sequenced using the Illumina NovaSeq 6000 platform (Illumina Inc., San Diego, CA, USA), following protocol provided by the manufacturer. Approximately 3.4 Gbp paired-end sequencing data were obtained for the *A. antiquum* sample. High-quality reads were used for *de novo* assembly with the low coverage whole genome sequencing (dnaLCW) method (Kim et al. 2015). In brief, the raw reads were trimmed using trimming tool, and then assembled into contigs using a CLC assembly tool (ver. 4.2.1, CLC Inc, Aarhus, Denmark). Using a previously reported chloroplast genome sequence of the *Asplenium* species as a reference, the assembled contigs with high similarity to the reference sequence were then extracted using MUMmer (Kurtz et al. 2004) and eventually assembled into a whole chloroplast genome sequence. Gene annotation on the draft chloroplast genome sequence of *A. antiquum* was performed using GeSeq (Tillich et al. 2017) and Artemis (Rutherford et al. 2000), and then manually curated. The schematic map of the chloroplast genome was generated using CPGView (Liu et al. 2023, http://www.1kmpg.cn/cpgview/). Finally, the complete chloroplast genome sequences and annotation of *A. antiquum* were submitted to GenBank under the accession number OR764773. The phylogenetic position of *A. antiquum* was infered with 19 representative species in the suborder Aspleniineae from NCBI GenBank (https://www.ncbi.nlm.nih.gov/genbank/), and *Arabidopsis thaliana* under Brassicaceae was included as an outgroup. A total of 74 chloroplast genome genes shared by all 20 species were used for phylogenetic reconstruction. Each CDS region of chloroplast genes were extracted by Feature Extract (Wernersson 2005), and then concatenated into a single matrix. The maximum-likelihood (ML) tree was reconstructed using MEGA 11, employing 1,000 bootstrap replicates, and the general time reversible model with gamma and invariable sites (GTR + G + I) model selected by MEGA 11 (Tamura et al. 2021).

## Results

**Figure 2.**
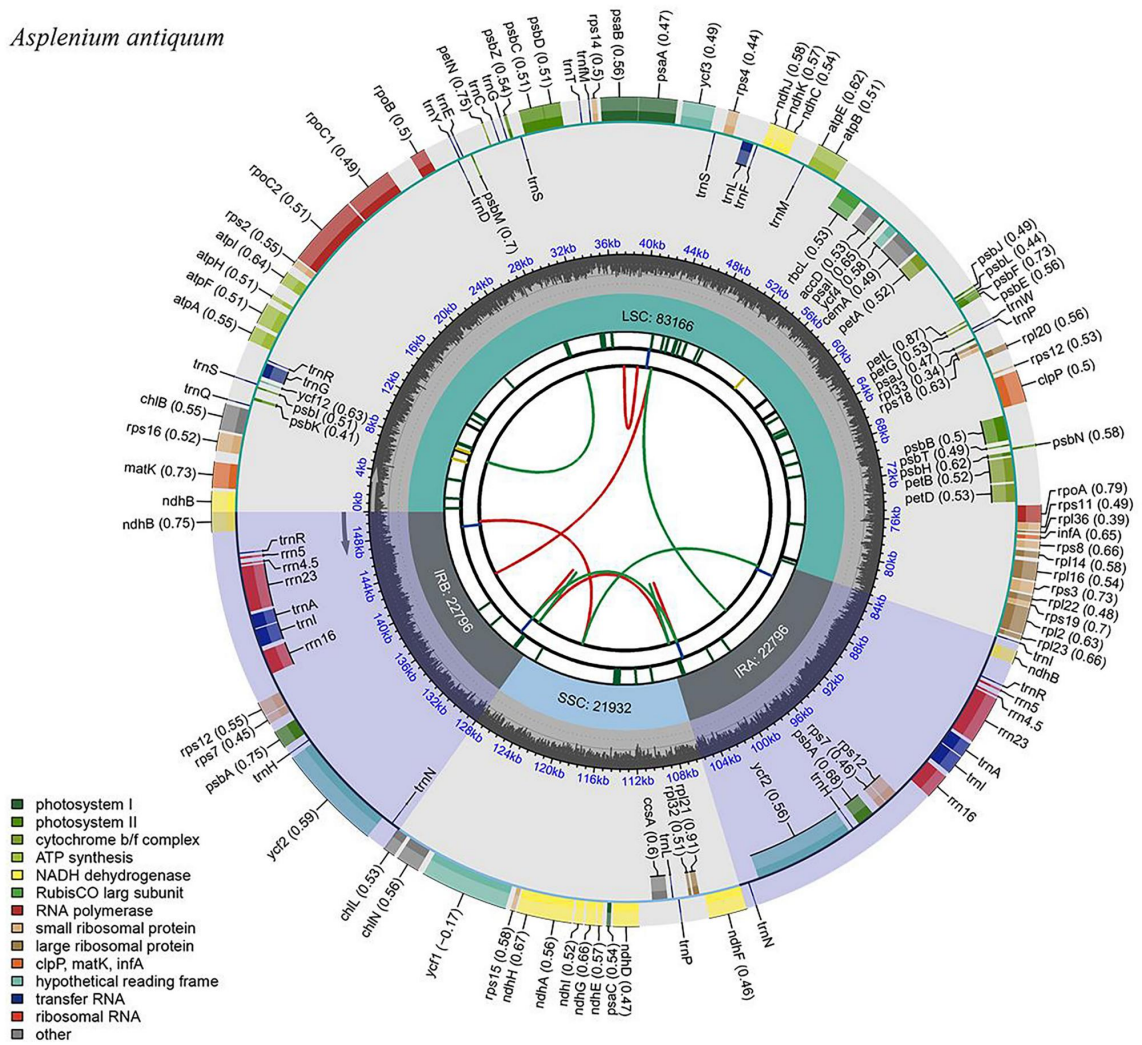
Circular map of the complete chloroplast genome *Asplenium antiquum*. The map contains six tracks delineating various features. The first track represents the dispersed repeat as a green and red arc. The second track represents a long tandem repeat as a blue bar. The third track represents a short tandem repeat in a combination of yellow, green, and black bars. On the fourth track, the quadripartite structure of chloroplast genome is represented: Large single-copy (LSC), small single-copy (SSC), and inverted repeats (IRA and IRB), respectively. The fifth track represents GC content in dark gray, and the sixth track represents chloroplast genes. Genes located on the inner and outer circles are transcribed in clockwise and counterclockwise directions, respectively. Optional codon usage bias is displayed in the parenthesis alongside the gene names. The functional classification of the genes is provided at the bottom-left corner of the figure.

The chloroplast genome of *A. antiquum* was 150,690 bp in length with 40.69% of GC content. The coverage depth of *A. antiquum* is shown in Figure S1. The genome structure was typical quadripartite, featuring two copies of inverted regions (IRa and IRb) with 22,796 bp each, a large single copy region (LSC) with 83,166 bp, and a small single copy (SSC) region with 21,932 bp in length (Figure 2). Excluding the overlapping genes in the IR region, the chloroplast genome contains 114 genes: 84 protein-coding genes, 26 tRNA genes, and 4 rRNA genes. Among the protein-coding genes, ten are cis-splicing genes; eight contain one intron and two genes (*ycf*3 and *clpP*) contain two introns (Figure S2). On the other hand, the *rps*12 gene has been recognized as a trans-splicing gene (Figure S3).

**Figure 3.**
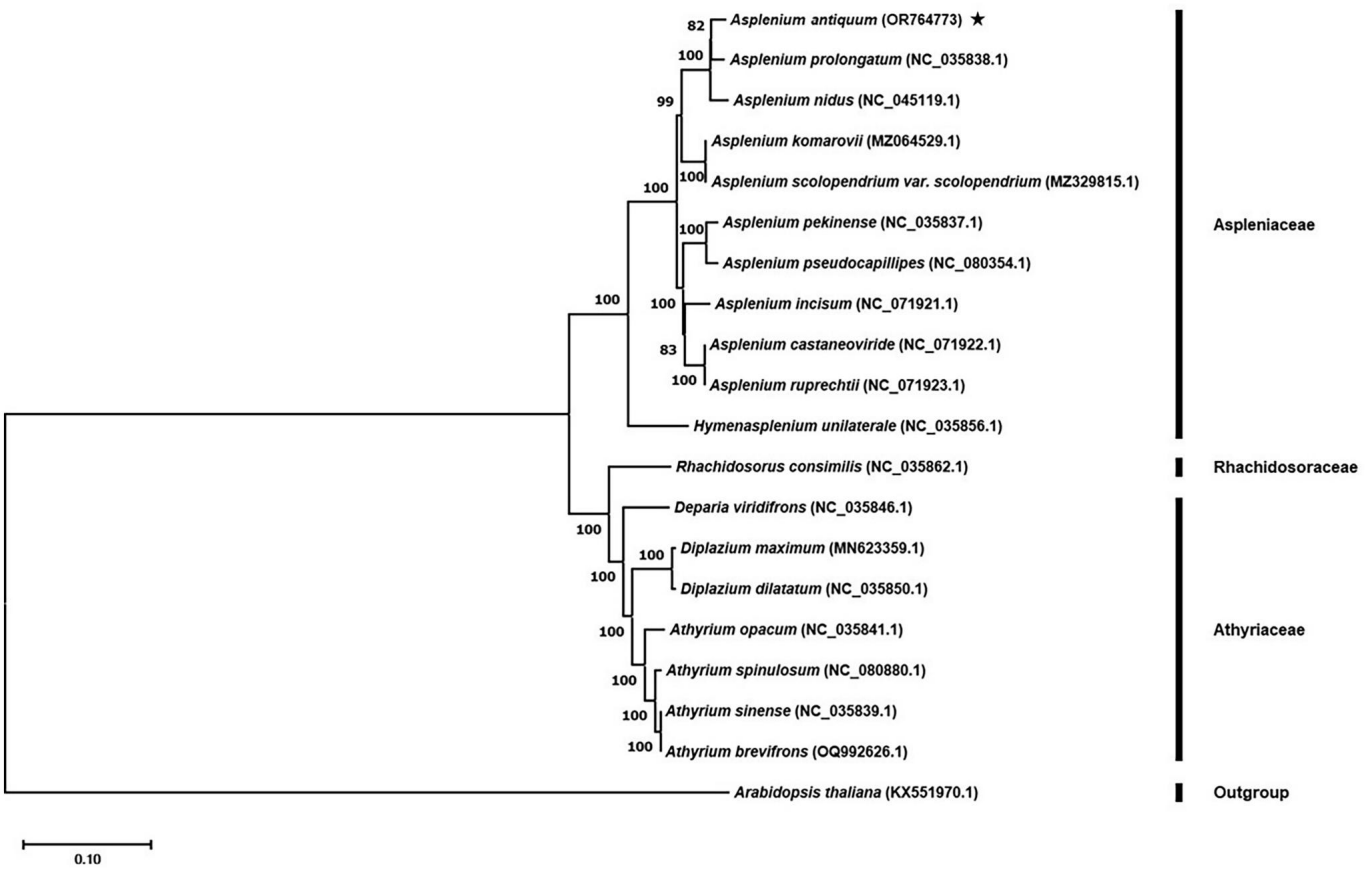
Phylogenetic analysis of suborder Aspleniineae based on 74 chloroplast genes from 20 species. The phylogenetic tree was constructed using the maximum-likelihood method (ML) (Tamura et al. 2021). Numbers at the node are the bootstrap supporting value calculated from the ML method. For the Aspleniaceae family, the following sequences were used: *Asplenium antiquum (*OR764773; this study), *Asplenium prolongatum* (NC_035838.1; Wei et al. 2017), *Asplenium nidus* (NC_045119.1; Cui et al. 2019), *Asplenium komarovii* (MZ064529.1), *Asplenium scolopendrium* var. *scolopendrium* (MZ329815.1), *Asplenium pekinense* (NC_035837.1; Wei et al. 2017), *Asplenium pseudocapillipes* (NC_080354.1), *Asplenium incisum* (NC_071921.1; Kim et al. 2023), *Asplenium castaneoviride* (NC_071922.1; Kim et al. 2023), *Asplenium ruprechtii* (NC_071923.1; Kim et al. 2023), *Hymenasplenium unilaterale* (NC_035856.1; Wei et al. 2017). For the Rhachidosoraceae family, *Rhachidosorus consimilis* (NC_035862.1; Wei et al. 2017) is included. For the Athyriaceae family, the following species are included: *Deparia viridifrons* (NC_035846.1; Wei et al. 2017), *diplazium maximum* (MN623359.1; Liu et al. 2020), *Diplazium dilatatum* (NC_035850.1; Wei et al. 2017), *Athyrium opacum* (NC_035841.1; Wei et al. 2017), *Athyrium spinulosum* (NC_080880.1), *Athyrium sinense* (NC_035839.1; Wei et al. 2017), *Athyrium brevifrons* (OQ992626.1). As for an outgroup, *Arabidopsis thaliana* (KX551970.1; Stadermann et al. 2016) from Brassicaceae is included.

The phylogenetic analysis with 19 representative species in the suborder Aspleniineae, including the newly completed *A. antiquum*, has illustrated that each representative species exhibits a monophyletic relationship with a bootstrap supporting value from 82 to 100% (Figure 3). Each species forms a distinct clade under the family level. The phylogenetic tree under the *Aspleniaceae* family, ten *Asplemum* species are grouped into three major clades (Figure 3). Among these clades, the newly reported *A. antiquum* (150,690 bp) exhibited a closer relationship with *A. proiongatum* (151,115 bp) while forming a sub-clade to *A. nidus* (156,173 bp). (Figure 3). *A. komarovii* and *A. scolopendrium* var. scolopendrium, with genome sizes of 149,393 and 149,379 bp, respectively, exhibited a 14 bp difference, forming a sister clade. Another clade comprises of *A. pekinense*, *A. pseudocapillipes, A. incisum, A. castaneoviride* and *A. ruprechtii*. Within this clade, *A. ruprechtii* and *A. castaneoviride* form a sister clade, with genome sizes of 153,066 and 153,071 bp, respectively, showing a 5 bp difference in genome size. These two species, in turn, form a sub-clade to with *A. incisum*, which has a genome size of 153,116 bp (Figure 3).

## Discussion and conclusions

The complete chloroplast genome of *A. antiquum* Makino was sequenced, assembled, and annotated for the first time. The phylogenetic analysis of *Asplenium* species exhibits monophyletic grouping, consistent with previously reported studies (Cui et al. 2019; Heo et al. 2021; Kim et al. 2023). Ten *Asplenium* species are grouped into three major clades, which appear to be separated by the size of the chloroplast genome. The newly reported *A. antiquum* is closely related to *A. prolongatum*, forming a sub-clade to *A. nidus*. The phylogenetic position of *A. antiquum* in the partial plastid sequence (Xu et al. 2020) demonstrated that *A. antiquum* is closely related to *A. nidus* than *A. prolongatum*. Such a finding suggests that complete chloroplast sequence could reveal a different phylogenetic postion for *Asplenium* species. More than 700 species of the genus *Asplenium* are present. However, the representation of *Asplenium* plant sequence in NCBI remains relatively limited as of the lastst up date on 18 April 2024. Therefore, further studies involving the complete assembly of *Asplenium* species are necessary. The chloroplast genome sequence of *A. antiquum* will provide the genetic information needed to understand the phylogenetic relationship within the genus *Asplenium*.

## Supplementary Material

Supplemental Material

## Data Availability

The data of this study is openly accession in GenBank of NCBI at https://www.ncbi.nim.nih.gov/ under the accession number OR764773. The associated BioProject, BioSample, and SRA numbers are PRJNA1033376, SAMN38031655 and SRS19312777.
