# Supplementary material for: The complete chloroplast genome sequence and phylogenetic analysis of Asplenium antiquum Makino 1929, an Endangered species in Korea
Source: Mitochondrial DNA B Resour. 2024 May 20;9(5):663–6. doi: 10.1080/23802359.2024.2356139 (PMC11107858; doi:10.1080/23802359.2024.2356139)

Supplementary Figure

**~~Figure S1. Sequencing and Coverage depth of~~ *~~Asplenium antiquum.~~***

**Figure S1. The coverage depth of the chloroplast genome of *Asplenium antiquum*.**


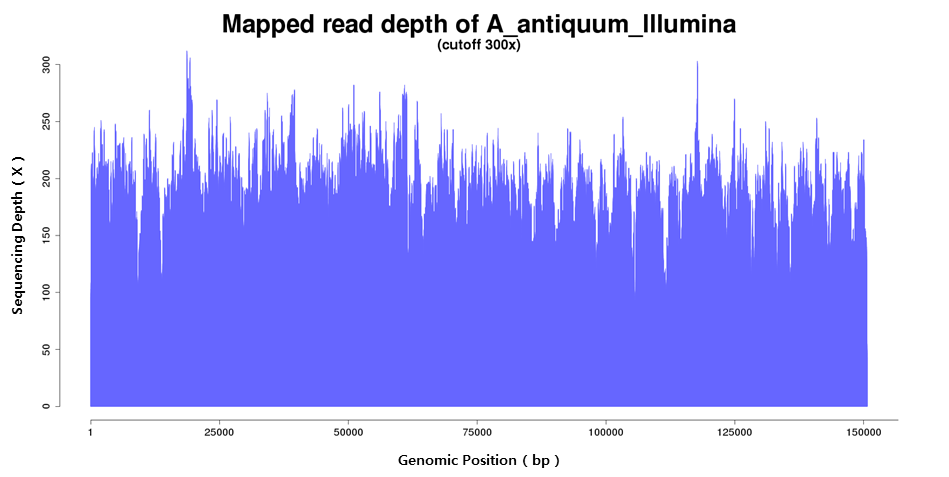


~~Figure S2. The structure of cis-splicing genes in~~ *~~Asplenium antiquum.~~* ~~The arrows indicate the sense direction of the genes.~~

**Figure S2. Schematic maps of cis-splicing genes in the chloroplast genome of *Asplenium antiquum* generated using CPGView.**


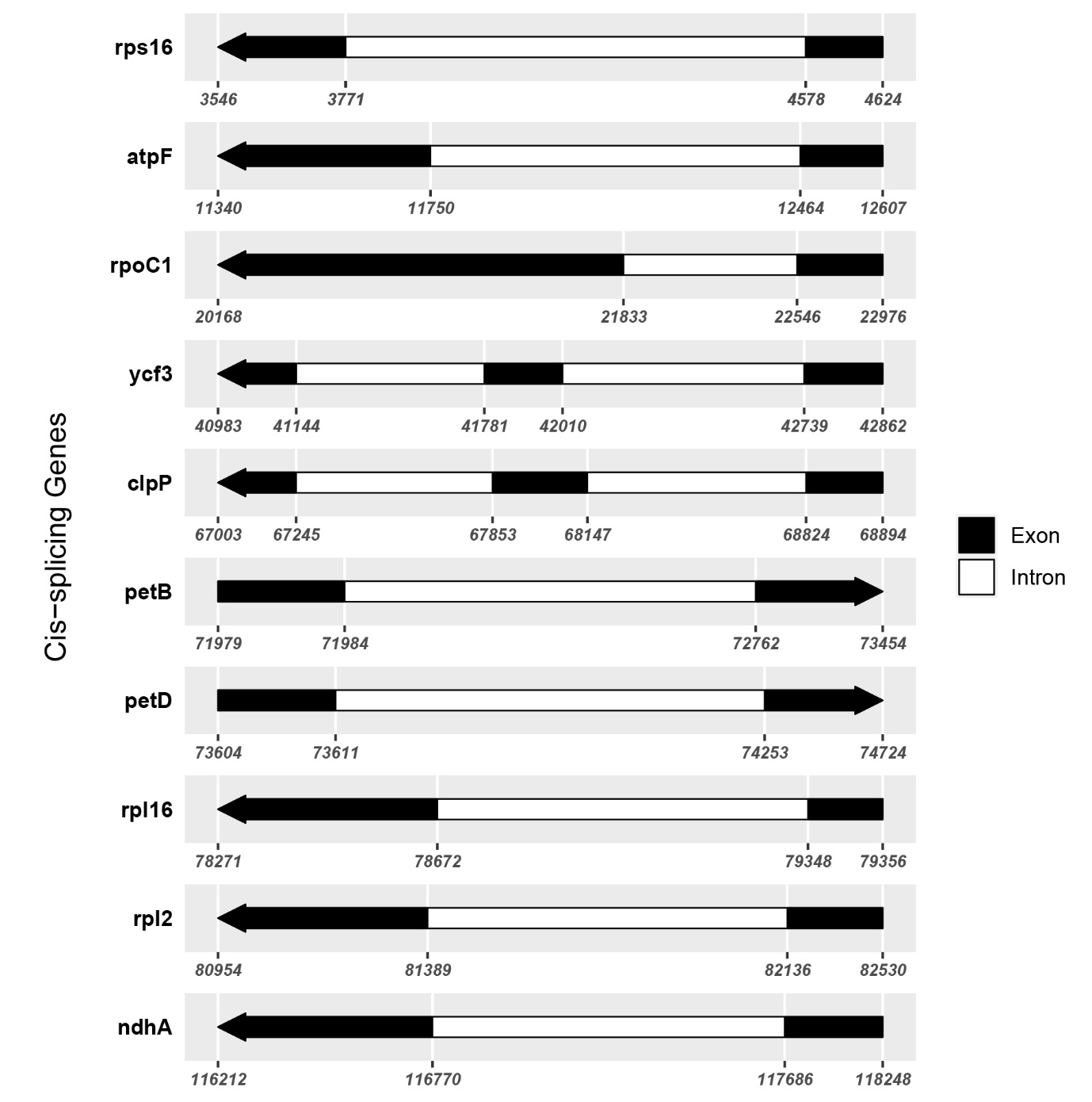


~~Figure S3. The structure of trans-splicing genes in~~ *~~Asplenium antiquum.~~* ~~The arrows indicate the sense direction of the genes~~.

**Figure S3. Schematic maps of the trans-spliced gene *rps12***


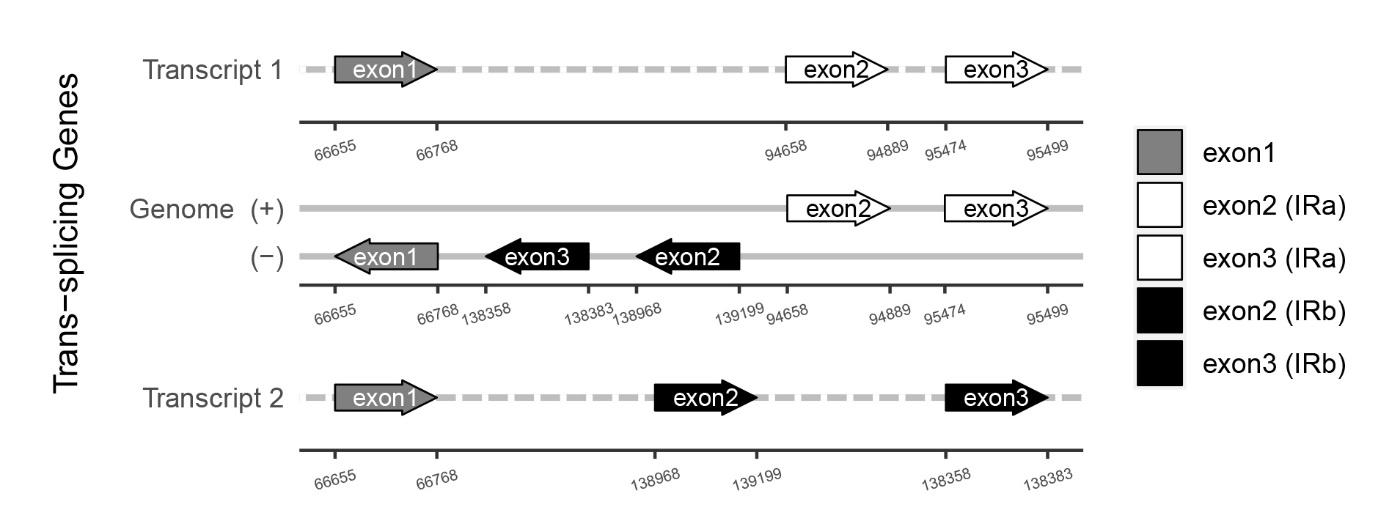

Supplement: Supplemental Material [file TMDN_A_2356139_SM9444.docx]
